# Wavy mucosal tear in the sigmoid colon

**DOI:** 10.1002/ccr3.2275

**Published:** 2019-08-07

**Authors:** Kentaro Tominaga, Junji Yokoyama, Kazunao Hayashi, Atsunori Tsuchiya, Shuji Terai

**Affiliations:** ^1^ Division of Gastroenterology and Hepatology, Graduate School of Medical and Dental Sciences Niigata University Niigata Japan

**Keywords:** collagen band, collagenous colitis, mucosal tear, proton‐pump inhibitor

## Abstract

Although linear mucosal tears have been reported, we found a relatively rare case of wavy mucosal tear in collagenous colitis. This may be related to the mutual weak adhesiveness between the epithelium and collagen band. This complication necessitates great care to be taken while performing colonoscopy in patients suspected of collagenous colitis.

## 1 QUESTION

**Figure 1:**
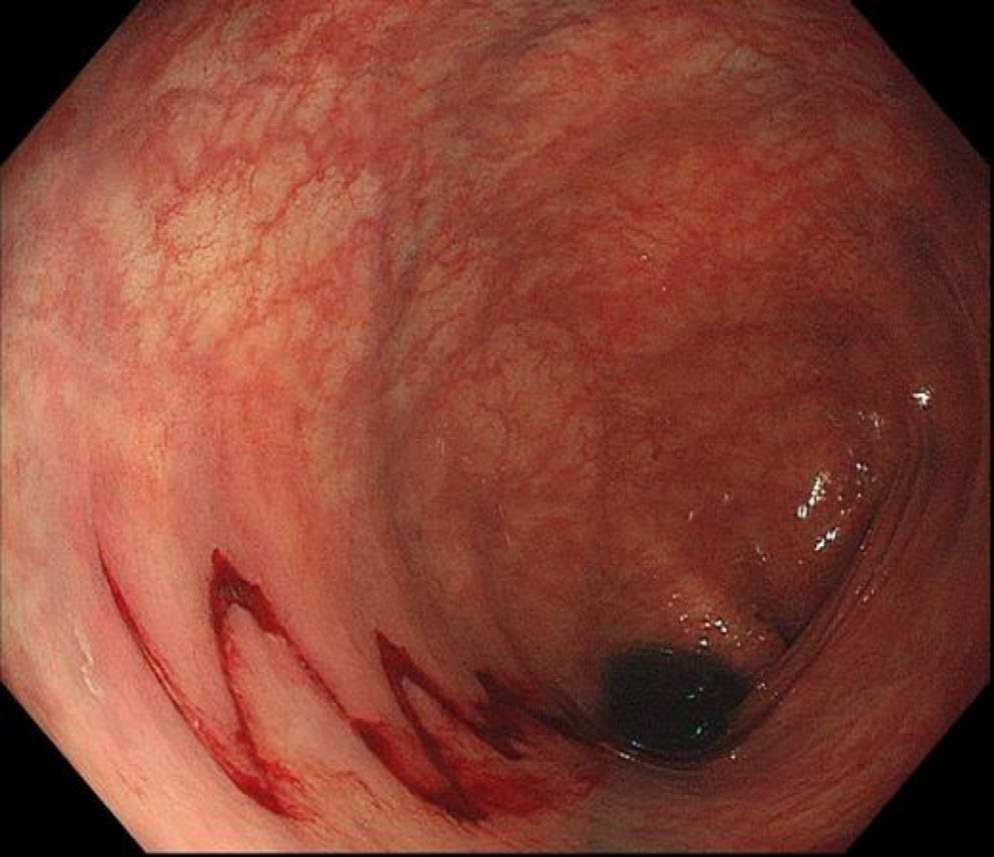
Colonoscopic findings in a patient of collagenous colitis. There is a wavy mucosal tear in the sigmoid colon

A 78‐year‐old woman was admitted with a three‐month history of watery, nonbloody diarrhea, and bodyweight loss. Physical examination and stool and blood test results were unremarkable. She had hypertension and osteoarthritis. Nonsteroidal anti‐inflammatory drugs and a proton‐pump inhibitor (lansoprazole) had been prescribed after surgery for osteoarthritis performed 4 months ago. During removal of the colonoscope, we found a 5‐cm‐long wavy mucosal tear in the sigmoid colon (Figure 1). What is the underlying disease of this patient?

## 2 ANSWER

**Figure 2:**
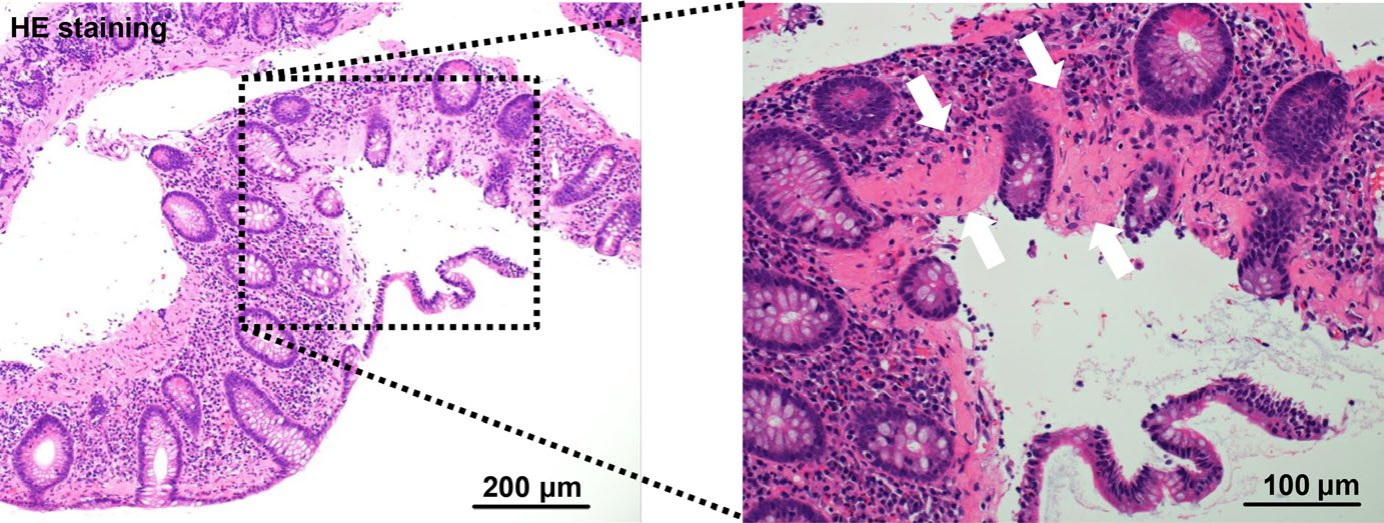
Histologic pictures (H&E stain) of a colonic biopsy specimen, showing irregular thickening of the subepithelial collagen band (white arrows) and inflammation of the lamina propria

**Figure 3:**
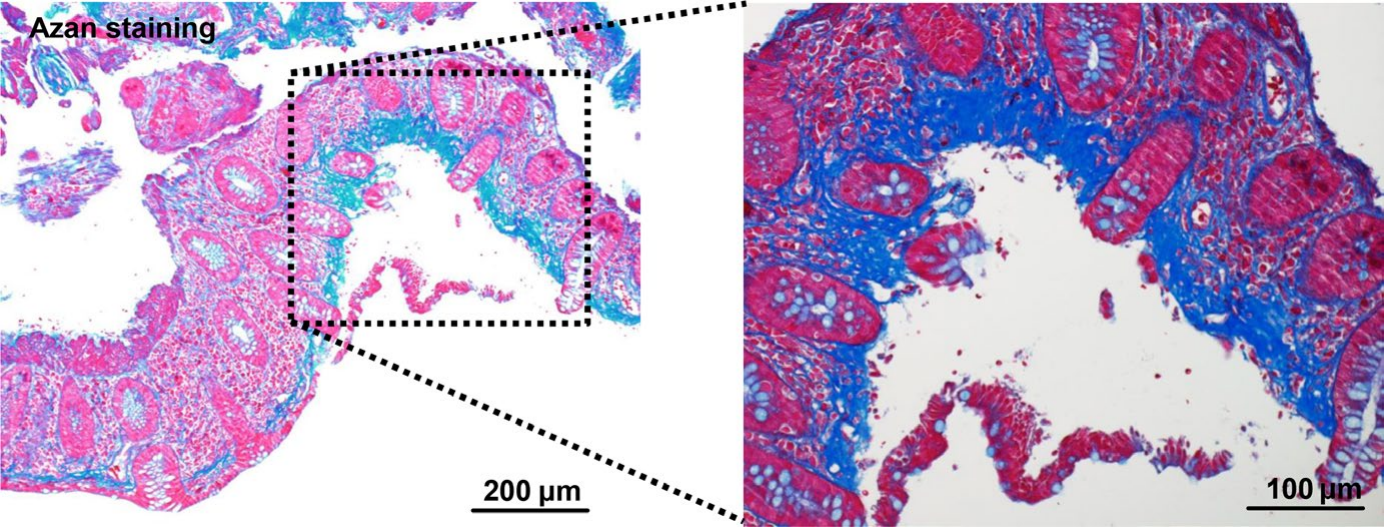
Histologic pictures (Azan stain) of a colonic biopsy specimen, showing typical findings of collagenous colitis. Diffuse thickening of the subepithelial collagen band with focal peeled surface epithelium can be seen

Collagenous colitis was diagnosed based on hematoxylin‐eosin stain (Figure 2) and Azan stain (Figure 3) of the specimens which showed irregular thickening of the subepithelial collagen band with focal peeled surface epithelium (Figure 2; white arrows). Discontinuing lansoprazole improved her diarrhea in 2 weeks. She was followed up for a year and she had no relapse.

Linear mucosal tears in collagenous colitis patients have been reported.^1^ However, wavy mucosal tears in collagenous colitis are relatively rare. This may be related to the mutual weak adhesiveness between the epithelium and collagen band,^2^ and the colonoscopy‐induced extension and shortening of the sigmoid colon may contribute to the wavy shape. Although the lesion itself appears limited to the mucosa in most cases, this complication necessitates great care to be taken while performing colonoscopy in patients suspected of collagenous colitis.

## CONFLICT OF INTEREST

The authors declare that they have no current financial arrangement or affiliation with any organization that may have a direct influence on their work. Informed consent was obtained from the patient for the publication of their information and imaging.

## AUTHOR CONTRIBUTIONS

All the authors made substantial contribution to the preparation of this manuscript and approved the final version for submission. KT and JY: drafted the manuscript. KH and AT: provided clinical support. ST: reviewed the manuscript carefully.
